# Smoking, alcohol consumption, drug abuse, and osteoporosis among older adults: a cross-sectional study on PERSIAN cohort study in Fasa

**DOI:** 10.1186/s12877-024-04678-y

**Published:** 2024-01-22

**Authors:** Zahra Khiyali, Vahid Rashedi, Ziba Tavacol, Azizallah Dehghan, Mostafa Bijani

**Affiliations:** 1 Iranian Research Center on Aging, Department of Aging, University of Social Welfare and Rehabilitation Sciences, Tehran, Iran; 2 Department of Medical-Surgical Nursing, School of Nursing, Fasa University of Medical Sciences, Fasa, Iran; 3 Noncommunicable Diseases Research Center (NCDRC), Fasa University of Medical Sciences, Fasa, Iran; 4 Department of Medical-Surgical Nursing, School of Nursing, Fasa University of Medical Sciences, Fasa, Iran

**Keywords:** Osteoporosis, Smoking, Alcohol, Drug abuse, Older adults, Cohort study

## Abstract

**Background:**

With increasing life expectancy and a growing population of older adults, the prevalence of osteoporosis has risen, resulting in a higher incidence of bone fractures, which necessitate extended treatment and specialized medical care. This study investigates the relationship between smoking, alcohol consumption, drug abuse, and osteoporosis among older adults in southern Iran, utilizing cohort data.

**Methods:**

This cross–sectional study is derived from the Fasa Adult Cohort Study (FACS), which included 10,133 individuals. From this cohort, we selected 1,631 older adults using census sampling methods. Our study aimed to explore the correlation between smoking, alcohol consumption, and drug abuse among older adults and the incidence of osteoporosis. We collected demographic information, nutritional indexes, medical history, glucocorticoid usage, and self-reported data on smoking, alcohol consumption, drug abuse, and osteoporosis through questionnaires. To investigate the relationship between smoking, alcohol, and drug use with osteoporosis while accounting for confounding factors, we employed logistic regression analysis.

**Results:**

The average age of the study participants was 64.09 ± 3.8 years, with a majority (898 (55.1%)) being female. Osteoporosis prevalence among the subjects was 25.20%. The results did not reveal a significant correlation between smoking, alcohol consumption, drug abuse, and osteoporosis (*p* > 0.05). Regression analysis identified gender, recent history of fractures within the past five years, history of using glucocorticoids, and physical activity as significant predictive risk factors for osteoporosis within the study population (*p* < 0.05).

**Conclusion:**

The study underscores the significance of addressing osteoporosis risk factors in older adults. Healthcare policymakers and administrators can use these findings to identify and mitigate influential factors contributing to osteoporosis in this demographic.

## Introduction

One of the biggest challenges the world faces today is an increasing number of older adults [1]. It is predicted that by 2050, the world’s older adults will have reached 2.1 billion [2]. People aged 60 years and above constitute 9.3% (approximately 7.5 million) of the total population of Iran, which number is predicted to rise to 11% by 2026 [3]. Aging is associated with chronic illnesses, disabilities, and cognitive impairment [4]. Hypertension, sleep disorders, indigestion, obesity, osteoporosis, and balance problems are among the health issues that correlate with old age [5–6]. Osteoporosis is a very serious problem among older adults and is reported to be most prevalent in Asia (24.3%) [5].

The most common metabolic bone disease, osteoporosis is caused by a lack of balance in the function of bone cells (imbalance between deterioration and production of bone), leading to a reduction in bone density and atrophy in bone tissues, which in turn increases the brittleness of bones and the risk of fractures [7]. In individuals with osteoporosis, the average bone mineral density (BMD) with a standard deviation of 2.5 is less than the healthy adults in a similar age and gender group (SD: T-score ≤ -2.5) [8]. Osteoporosis causes acute and chronic pain, increases the risk of hospitalization, and reduces activities of daily living (ADL) in older adults. Fractures because of osteoporosis are known as the most serious clinical consequences of the disease, which cause lengthy treatments and special medical management and are associated with increased mortality and a considerable socio-economic burden [9–12]. Complex interactions between genetic and environmental factors have been reported which help a better understanding of the pathophysiology of osteoporosis. These factors include smoking, alcohol consumption, lack of physical activity, weight change, inadequate intake of nutrients, especially calcium and vitamin D, history of fractures, use of, hormonal factors, genetic factors, and being female [13–15]. Research shows that smoking can disrupt the cycle of bone growth, which reduces bone mass and density and increases the risk of osteoporosis [16–17]. Tobacco smoke indirectly impacts bone mass by affecting body weight changes, the PTH-vitamin D axis, adrenal hormones, sex hormones, and elevation of oxidative stress in bone tissues. Also tobacco smoke has a direct impact on bone mass [18]. Alcohol consumption is linked to an increased rate of fractures and accompanying consequences. However, little is known about the complicated effects of alcohol on bone tissues. More research into the long-term effects of smoking and alcohol use on osteoporosis is recommended [8].

Among older adults, the most commonly abused drugs are alcohol, smoking, hashish, and prescription drugs, including opioids and benzodiazepines [18]. The results of a 2018 study in the U.S. showed that, in the course of 12 months, the rate of abuse of alcohol, smoking, hashish, and opioids (prescription opioids included) by individuals aged 65 years and above was 43%, 14%, 4.1%, and 1.3% respectively [19]. A study by Ahmadi et al. (2022) reported that 11.3% of the older adults in Tehran abused substances: 3.74% and 83.20% were addicted to opium and alcohol respectively [20]. Some studies show that opium with inhibiting Gonadotropin-releasing hormone (GnRH) and the ability of Inhibition of osteoblasts؛ may be associated with increased risk of osteoporosis [21]. Gozashti et al. and Li et al. indicated that opium addicts are more vulnerable to the occurrence of BMD loss compared with non-addicts [22–23]. According to a meta-analysis by Godos et al., excessive consumption of alcohol correlates with a higher risk of f fractures because of osteoporosis and pelvic fractures [24]. Most of these studies suffer from ignoring the role of potential confounders such as physical activity and dietary factors in their analysis.

Thus, the prevention of osteoporosis and resulting fractures by identifying and understanding the controllable risk factors is essential. Because of the increase in the number of older adults, drug abuse in this population, the socioeconomic burden of osteoporosis on the patients and their society, and the lack of a study of this type in Fasa, the present study investigated the relationship between smoking, alcohol consumption, and drug abuse and osteoporosis in the older adults in a PERSIAN cohort study in Fasa.

## Methods

The present study is an analytical cross-sectional study that was conducted using the data of the first phase of the PERSIAN Fasa adult cohort. The first phase of the study lasted from 2014 to 2016. This study was performed on 1,631 older adults referring to the Fasa Adult Cohort Study (FACS). FACS is a branch of PERSIAN Cohort (Prospective Epidemiological Research Studies in Iran) initiated in 2014. The Fasa Adult Cohort (FACS) was started in November 2014 to evaluate the risk factors associated with the most common non-communicable diseases in the residents of the rural area of Fasa. This study is a population-based longitudinal survey with a total follow-up period of 15 years. In this cohort were included, all adults over 35 years of age who had lived in rural areas for at least 9 months. However, subjects with physical or mental disabilities who could not complete the measurements or answer the questions were excluded from the study. Overall, 10,133 subjects were included in the original cohort study. Out of this number, 1631 people were over 60 years old, and these people were considered for the current study. In this study, the information from the first phase of the Persian Fasa cohort was used. In this cohort study, demographic information including age, gender, marital status, education level, number of pregnancies and abortions, history of bone fractures in the last 5 years, history of tubectomy and hysterectomy, history of diseases (diabetes, blood pressure, cardiovascular) were collected through Personal interview and self-report. Physical activity was measured with a 20-question questionnaire that was designed to measure the activity of Iranians in rural areas. The time of each activity (in hours) per day was multiplied by the MET value of that activity, and the physical activity of each person was recorded as MET/day. The complete data collection sheet and questionnaire are available at ncdrc.fums.ac.ir. Weight was measured with a digital scale with an accuracy of 0.1 kg. Height was measured using a stadiometer with an accuracy of 0.1 cm. BMI was calculated by dividing weight in kilograms by the square of height in meters (kg/m²). To record the medications taken, including the history of using glucocorticoids, the participants were asked to bring their medications with them at the time of the interview to record the medication history as accurately as possible. Smoking, alcohol use, and drug abuse were recorded by self-report and through the answer to the question “whether they have used smoking, alcohol, or drugs in the last 12 months.” Osteoporosis was recorded by self-report and by answering the question “Has the doctor told you that you have osteoporosis?“. Nutritional indicators (vitamin D, vitamin E, calcium, phosphorus, calorie intake) were measured through the Food Frequency Questionnaire (FFQ). The cohort study was approved by the Ethics Committee of the Fasa University of Medical Sciences (IR.FUMS.REC.1395.177) and the study was conducted following the Helsinki Declaration and Iranian national guidelines for ethics in research. Details of the FACS have been described extensively elsewhere [25, 26].

After acquiring a research ethics permit and a license from the Department of Research, the researchers extracted needed information from the Fasa cohort. The extracted data were compared into two groups based on whether they had or did not have osteoporosis.

The collected data were analyzed using SPSS 23 (Armonk, NY: IBM Corp) and descriptive and inferential statistics, including t-test, chi-square, analysis of variance, and logistic regression. Logistic regression was used to investigate the relationship between smoking, alcohol, and drugs with osteoporosis by removing the effect of confounding factors. P-values of less than 0.05 were considered significant.

### Ethical considerations

All the participants gave written informed consent to participate in the study. The present study was conducted in terms of the principles of the revised Declaration of Helsinki, which is a statement of ethical principles that directs physicians and other participants in medical research involving human subjects. The participants were assured about their anonymity and confidentiality of their information. Moreover, the study was approved by the Institutional Research Ethics Committee of Fasa University of Medical Sciences, Fasa, Iran (ethical code: IR.FUMS.REC.1401.246).

## Results

In the present study, 1,631 individuals were studied. The mean age of the subjects was 64.09 ± 3.8 years, with most of the subjects (898 (55.1%)) being female. All the female subjects had experienced menopause, and the number of pregnancies and abortions among them were 8.98 ± 3.25 and 0.73 ± 1.08 respectively. The body mass index (BMI) of the subjects was 25.09 ± 4.49 (kg/m²),their calorie intake was 2888.44 ± 1177.85 (kcal), and their physical activity was 39.82 ± 10.83 (MET/day). The prevalence of osteoporosis among them was found to be 25.2%. Table 1 shows the other demographic characteristics of the subjects.

**Table 1:** Frequency distribution of the study population’s demographic characteristics

Variable		n	%
Gender	Male	733	44.90
	Female	898	55.10
Marital status	Unmarried	6	0.40
	Married	1625	99.60
Education	Uneducated	1111	68.10
	Primary school	350	21.50
	Junior high-school	149	9.10
	High-school and above	21	1.30
Osteoporosis	Positive	411	25.20
	Negative	1220	74.80
History of bone fractures	Positive	632	38.70
	Negative	999	61.30
Smoking	Positive	477	29.20
	Negative	1154	70.80
Drug abuse	Positive	235	14.40
	Negative	1396	75.60
Alcohol consumption	Positive	26	1.60
	Negative	1605	98.40
Diabetes	Positive	1276	78.20
	Negative	355	21.80
Hypertension	Positive	713	43.70
	Negative	918	56.30
Cardiovascular diseases	Positive	400	24.50
	Negative	1231	75.50
History of using glucocorticoids	Positive	30	1.80
	Negative	1601	98.20

The results showed that the rate of osteoporosis was higher in females than the males. Also, the variables of smoking, alcohol consumption, and drug abuse were significant in both the group with osteoporosis and the group without osteoporosis. Table 2 shows the basic characteristics of the subjects with and the subjects without osteoporosis. The logistic regression results showed that the variables of of gender, physical activity, history of a bone fracture in the past five years, and history of using glucocorticoids were predicting risk factors of osteoporosis in the older adults who were studied. Being female, having a history of a bone fracture in the past five years, and using prednisolone increased the risk of osteoporosis by 7.22%, 2.19%, and 4.16% respectively. On the other hand, physical activity was found to be a protective factor against osteoporosis (Table 3).

**Table 2:** A comparison between the basic characteristics of the subjects with and without osteoporosis

Variable		Osteoporosis (−)	Osteoporosis (+)	***p***-value
Age (mean ± SD)		64.05 ± 4.00	64.22 ± 3.48	0.42*
BMI (kg/m^2^) (mean ± SD)		24.80 ± 4.34	25.93 ± 4.81	< 0.001*
Number of pregnancies (mean ± SD)		8.89 ± 3.23	9.10 ± 3.29	0.35*
Number of abortions (mean ± SD)		1.06 ± 0.69	1.09 ± 0.79	0.18*
Calorie intake (mean ± SD)		2947.53 ± 1214.02	2713.75 ± 1045.77	< 0.001*
Physical activity (MET/day mean ± SD)		40.93 ± 11.50	36.55 ± 7.65	< 0.001*
Vitamin D (mean ± SD) (nmol/L)		53.24 ± 45.09	48.75 ± 37.74	0.07*
Vitamin K (nmol/L) (mean ± SD)		137.28 ± 3.93	201.65 ± 161.68	0.88*
Calcium (mmol/L mean ± SD)		1425.25 ± 695.07	1390.80 ± 658.59	0.37*
phosphorus (mmol/L mean ± SD)		1404.00 ± 581.70	1344.92 ± 545.48	0.07*
Gender N (%)	Male	675 (92.1%)	58 (7.9%)	< 0.001**
	Female	545 (60.7%)	353 (39.3%)	
History of a bone fracture in the past five years N (%)	Positive	447 (70.7%)	185 (29.3%)	0.003**
	Negative	773 (77.4%)	226 (22.60%)	
History of tubectomy N (%)	Positive	177 (62.30%)	107 (37.70%)	0.49**
	Negative	368 (59.90%)	246 (40.10%)	
History of hysterectomy N (%)	Positive	44 (54.30%)	37 (45.70%)	0.21**
	Negative	501 (61.30%)	316 (38.70%)	
Smoking N (%)	Positive	830 (71.90%)	324 (28.10%)	< 0.001**
	Negative	390 (81.80%)	87 (18.20%)	
Drug abuse N (%)	Positive	1010 (72.30%)	386 (27.70%)	< 0.001**
	Negative	210 (89.40%)	25 (10.60%)	
Alcohol consumption N (%)	Positive	25 (96.20%)	1 (3.80%)	0.01**
	Negative	1195 (74.5%)	410 (25.50%)	
Diabetes N (%)	Positive	229 (50.64%)	126 (50.35%)	< 0.001**
	Negative	991 (70.77%)	285 (30.22%)	
Hypertension N (%)	Positive	483 (67.70%)	230 (32.30%)	< 0.001**
	Negative	737 (80.30%)	181 (19.70%)	
Cardiovascular diseases N (%)	Positive	269 (67.30%)	131 (32.80%)	< 0.001**
	Negative	951 (77.30%)	280 (22.70%)	
History of using glucocorticoids N (%)	Positive	13 (43.30%)	17 (56.70%)	< 0.001**
	Negative	1207 (75.40%)	394 (24.60%)	

*Independent t-test

**chi-square test

BMI: Body Mass Index

**Table 3:** Logistic regression results for the predicting risk factors of osteoporosis in the older adults under study

Variable	B	***p***-value	OR	CI for OR 95%	
				Upper	Lower
Gender	1.97	< 0.001	7.22	1.02	5.05
BMI*	0.21	0.13	1.02	1.05	0.99
Number of abortions	0.08	0.22	1.08	1.23	0.95
history of a bone fracture in the past five years	0.78	< 0.001	2.19	3.28	1.46
Diabetes	0.17	0.27	1.19	1.63	0.87
Hypertension	0.16	0.28	1.17	1.58	0.87
Smoking	-0.03	0.86	0.96	1.43	0.65
Drug abuse	0.61	0.37	1.84	7.02	0.48
Calorie intake	0.0001	0.13	1.00	1.00	1.00
Physical activity	-0.03	0.002	0.86	0.98	0.94
Hysterectomy	0.29	0.22	1.34	2.15	0.83
Cardiovascular diseases	0.11	0.49	1.11	1.53	0.81
Vitamin D	0.0001	0.93	1.00	1.00	0.99
Phosphorus	0.0001	0.71	1.00	1.00	1.00
History of using glucocorticoids	1.42	0.004	4.16	10.97	1.57

*BMI: Body Mass Index

## Discussion

The findings of the study showed the total prevalence of osteoporosis among older adults to be 25.20%. The high rate of osteoporosis and fractures associated with it poses a serious challenge to not only healthcare authorities but the patients and their families and the whole society [27]. A study of 2,425 older adults in Bushehr by Fahimfar et al. found the prevalence of osteoporosis to be 41.50% [28]. A similar study of old patients in Chinese hospitals by Li et al. (2019) showed that 62.80% of patients aged over 60 years suffered from osteoporosis [29]. Differences in sample size and study settings can explain the difference between the rates of the disease as reported by the two studies; so that in this study, most of the subjects were women and community- dwelling. In a study by Zhang et al., the prevalence of osteoporosis in older adults aged above 70 years was reported to be 39.5%, which is greater than the rate determined by the present study [30]. This discrepancy can be attributed to differences between the ages of the subjects: research shows that primary osteoporosis is more common among women who have experienced menopause and men and women who are aged 70 years and above [5, 31].

The present study’s findings showed that the variables of smoking and drug abuse weren’t identified as predicting risk factors of osteoporosis in older adults. Similarly, a study by Mousavi et al. reported that smoking did not correlate with osteoporosis [32].

However, other studies verify that there is a correlation between the above-mentioned variables and osteoporosis. For example, studies of older adults conducted by Hayder et al. [17] and Hou et al. [33] found that smoking impacted osteoporosis. Also, Weng et al. [34] reported that smoking leads to a reduction in bone density and is, therefore, an independent risk factor for osteoporosis. Likewise, the results of a study by Yang et al. [7] demonstrated that smoking has a great impact on osteoporosis, especially in males. Wang et al. [35] reported that regular smoking significantly increases the risk of osteoporosis and osteopenia. Similarly, Zamani et al. [36] and Irani et al. [37] found heavy smoking (over 20 cigarettes a day) to be a major risk factor for osteoporosis. A study of older adults by Marques et al. [38] showed that, after the confounding variables were moderated, current and previous smoking harmed the health of bones and current smoking in particular can aggravate loss of bone density in older ages. As for drug abuse, most studies have indicated a correlation between opioids and osteoporosis [21–23, 39]. In general, the discrepancy between the findings of the present study and the above studies can be explained, by smoking, some of the older adults might have refused to mention that they smoked. As for drug abuse, This may be because most studies have not adjusted the association of opium and BMD based on potential confounders, while we, in our adjusted analysis, considered the role of smoking, BMI, nutritional indexes, history of diseases, history of using glucocorticoids and physical exercise simultaneously which may provide more valid results. Moreover, more than half of the subjects in the present study were postmenopausal females and the confounding effect of menopause can explain the insignificance of the impact of smoking and drug abuse on osteoporosis in the subjects who were studied.

Based on the results of the present study, alcohol consumption was significantly reported in people with osteoporosis compared to healthy people. A meta-analysis by Cheraghi et al. [40] pointed to a positive correlation between alcohol consumption and osteoporosis. There is evidence of a correlation between excessive consumption of alcohol and a higher risk of fractures due to osteoporosis and pelvic fractures [24, 41]. On the other hand, several studies have found that low-to-moderate consumption of alcohol may have protective effects on the health of bones in female adults; yet, the impact of alcohol in smaller doses is unknown as the BMD of even light drinkers was found to be higher than that of non-drinkers [24, 42]. However, based on the results of the present study, alcohol consumption was significantly reported in people with osteoporosis compared to healthy people. However, because of the very low prevalence of alcohol in the present study, which may be due to the lack of routinely consumed in the religious and cultural context of the city of Fasa, especially among women, or the lack of reporting of its consumption by the participants, it was not included in the regression model.

In the present study, the variables of gender, history of a bone fracture in the past five years, and history of using glucocorticoids were identified as predicting risk factors of osteoporosis in older adults. On a similar note, in a study by Mousavi et al. [32], gender, use of cortisone, and having a history of a bone fracture after the age of 40 were found to have a significant correlation with bone density. As with the present study, many other studies have confirmed the correlation between being female and an increased risk of osteoporosis [5, 30, 43, 44]. Being female is an independent risk factor of osteoporosis among older adults: osteoporosis is more common among women than men. This increase may be associated with a reduction in estrogen levels following menopause in women [30]. Wang et al. [35] found having a history of a bone fracture and greater risk of osteoporosis to be correlated. Studies refer to having a history of bone fracture as the primary risk factor for osteoporosis in men [45]. As for using cortisone, Schmidt et al. [46] reported that the use of glucocorticoids can increase the risk of osteoporosis. Glucocorticoids are effective immunosuppressive drugs with many uses. These drugs manifest a predictable effect on bones which is characterized by a rapid reduction in bone density in the first 3 to 6 months after administration, followed by a gradual decrease in bone formation. Therefore, the dose and length of use of glucocorticoids should be carefully controlled for all patients. Patients should be counseled about lifestyle-related measures they can take to preserve their bone density, including nutrition and weight-bearing exercises [47]. Previous studies confirm that the use of glucocorticoids is a common secondary cause of osteoporosis among adults, especially patients with rheumatoid arthritis, which is consistent with the findings of the present study [48, 49].

In the present study, regular physical exercise was identified as a protective factor against osteoporosis. Similarly, previous studies have found playing sports and regular physical exercise as important protective factors against osteoporosis [8, 50, 51]. According to a systematic review, physical activities probably have significant clinical advantages in preventing osteoporosis in older adults, especially activities that include a variety of exercises and resistance training performed regularly (at least 2 to 3 times a week) for + 60 min [52]. Studies show that physical activities can not only prevent osteoporosis, but also help manage this condition in individuals who have osteoporosis: physical activities and safe exercise can promote bone density, reduce the risk of falling and fractures, improve posture, help manage the symptoms of spinal fractures, and minimize the potential hazards of the disease in individuals with osteoporosis [53].

## Limitations

The Cross-sectional nature of our data constrains our ability to establish causal relationships between the variables of interest. Future research should adopt longitudinal designs and prospective cohort methods in diverse regions of Iran and other countries to enhance our understanding. Another limitation of the current study was the self-reported nature of osteoporosis, which may ignore some positive cases and underreport the reality. Therefore, it is suggested that osteoporosis be measured and recorded based on laboratory (lab) test and Bone Mineral Densitometry (BMD).

## Conclusion

This study has pinpointed several key factors that predict the risk of osteoporosis among older adults. Specifically, gender, a history of bone fractures in the past five years, a history of using glucocorticoids, and physical activity emerged as significant predictors. Notably, smoking, alcohol consumption, and drug abuse did not exhibit a significant correlation with osteoporosis, although it’s essential to approach these findings cautiously. To gain further insights, prospective cohort studies are strongly recommended. This research underscores the importance of recognizing and addressing these risk factors to aid in the prevention and management of osteoporosis in older adults.

## Data Availability

The datasets generated and/or analysed during the current study are not publicly available due to the necessity to ensure participant confidentiality policies and laws of the country but are available from the corresponding author on reasonable request.

## Abbreviations

FACS: Fasa Adalt Cohort Study

## References

[1] Barsukov VN (2019). From the demographic dividend to population ageing: world trends in the systemwide transition. Economic and Social Changes: Facts Trends Forecast.

[2] World Health Organization, Ageing. and Health; 2021. https://www.who.int/news-room/fact-sheets/detail/ageing-and-health. Accessed March 18, 2022.

[3] Zanjari N, Sadeghi R. Measuring of Older Adults’ Well-Being in Iranian Provinces Using Age Watch Index. Salmand: Iranian Journal of Ageing.2022;16(4).1–10 http://salmandj.uswr.ac.ir/article-1-2009-fa.html.

[4] Khoddam H, Eshkevarlaji S, Nomali M, Modanloo M, Keshtkar AA (2019). Prevalence of malnutrition among older adults people in Iran: protocol for a systematic review and meta-analysis. JMIR Res Protoc.

[5] Salari N, Ghasemi H, Mohammadi L, Rabieenia E, Shohaimi S, Mohammadi M (2021). The global prevalence of osteoporosis in the world: a comprehensive systematic review and meta-analysis. J Orthop Surg Res.

[6] Nabavi SH, Hatami ST, Norouzi F, Gerivani Z, Hatami SE, Monadi Ziarat H, Delbari A (2016). Prevalence of fall and its related factors among older people in bojnurd in 2015. Iran J Ageing.

[7] Aibar-Almazán A, Voltes-Martínez A, Castellote-Caballero Y, Afanador-Restrepo DF, Carcelén-Fraile MDC, López-Ruiz E (2022). Current status of the diagnosis and management of osteoporosis. Int J Mol Sci.

[8] Yang, Jerry CY, Huang W (2021). Effects of sex, tobacco smoking, and alcohol consumption osteoporosis development: evidence from Taiwan biobank participants. Tob Induc Dis.

[9] Borgström F, Karlsson L, Ortsäter G, Norton N, Halbout P, Cooper C (2020). Fragility fracture in Europe:burden, management and opportunities. Arch Osteoporos.

[10] Johnston CB, Dagar M (2020). Osteoporosis in older adults. Med Clin North Am.

[11] Mcarthur C, Lee A, Alrob HA (2022). An update of the prevalence of osteoporosis, fracture risk factors, and medication use among community-dwelling older adults: results from the Canadian longitudinal study on aging (CLSA). Arch Osteoporos.

[12] Pouresmaeili F, Kamalidehghan B, Kamarehei M, Goh YM (2018). A comprehensive overview on osteoporosis and its risk factors. Ther Clin Risk Manag.

[13] Aslam H, Holloway-Kew KL, Mohebbi M, Jacka FN, Pasco JA, 15 (2019). Association between dairy intake and fracture in an Australian-based cohort of women: a prospective study. BMJ Οpen.

[14] Doosti-Irani A, Ghafari M, Cheraghi Z (2018). The high prevalence of osteoporosis as a preventable disease: the need for Greater Attention to Prevention Programs in Iran. Iran J Public Health.

[15] Askari M, Lotfi MH, Azimi M, Ostovarfar M, Fallahzadeh H, Mehrabbeik A, Hamedi A (2022). Risk factors of osteoporosis in females: a hospital-based case-control study, Yazd, Iran. Iran J Public Health.

[16] Al-Bashaireh AM, Haddad LG, Weaver M, Chengguo X, Kelly DL, Yoon S (2018). The Effect of Tobacco Smoking on Bone Mass: an overview of pathophysiologic mechanisms. J Osteoporos.

[17] Hayder DM, Alwan FJ, Gadhban AQ (2022). The effect of smoking on osteoporosis. Web of Scientist: International Scientific Research Journal.

[18] Hayek E (2022). Prevention Strategies of Alcohol and Substance Use disorders in older adults. Clin Geriatr Med.

[19] Center for Behavioral Health Statistics and Quality (2019). 2018 National Survey on Drug Use and Health: detailed tables.

[20] Ahmadi S, Mohaqeqi Kamal M, Basakha SH (2022). Substance use among Iranian elderly: opium, a substance for all seasons. J Subst Use.

[21] Sanjari M, Yarmohammadi H, Fahimfar N (2022). The association of opioid consumption and osteoporosis in old men: Bushehr Elderly Health (BEH) program. Arch Osteoporos.

[22] Gozashti MH, Shahesmaeili A, Amini Zadeh N (2011). Is opium addiction a risk factor for bone loss?. Iran Red Crescent Med J.

[23] Li L, Setoguchi S, Cabral H, Jick S (2013). Opioid use for noncancer pain and risk of fracture in adults: a nested case-control study using the general practice research database. Am J Epidemiol.

[24] Godos JF, Giampieri E, Chisari A, Micek N, Paladino TY (2022). Alcohol consumption, bone mineral density, and risk of osteoporotic fractures: a dose-response meta-analysis. Int J Environ Res Public Health.

[25] Farjam M, Bahrami H, Bahramali E, Jamshidi J, Askari A, Zakeri H (2016). A cohort study protocol to analyze the predisposing factors to common chronic non-communicable diseases in rural areas: Fasa Cohort Study. BMC Public Health.

[26] Homayounfar R, Farjam M, Bahramali E, Sharafi M (2023). Cohort Profile: the Fasa adults Cohort Study (FACS): a prospective study of non-communicable diseases risks. Int J Epidemiol.

[27] Park SB, Kim J, Jeong JH, Lee JK, Chin DK, Chung CK (2016). Prevalence and incidence of osteoporosis and osteoporotic vertebral fracture in Korea: nationwide epidemiological study focusing on diferences in socioeconomic status. Spine.

[28] Fahimfar N et al. Prevalence of osteoporosis among the elderly population of Iran. Archives of osteoporosis, 2021;16 (2021): 1–10. 10.1007/s11657-020-00872-8 33475880

[29] Li H, Tianbao S, Dongmei H (2023). Risk factors of osteoporosis in elderly inpatients: a cross-sectional single-centre study. Front Aging.

[30] Zhang Q, Cai W, Wang G, Shen X (2020). Prevalence and contributing factors of osteoporosis in the elderly over 70 years old: an epidemiological study of several community health centers in Shanghai. Ann Palliat Med.

[31] Marcucci G, Brandi ML (2015). Rare causes of osteoporosis. Clin Cases Miner Bone Metab.

[32] Mousavi M, Pakzad B, Ebrahimian M (2022). Investigating the decreased bone density prevalence in people with common risk factors referred to Al-Zahra Educational-Medical Center in Isfahan City years of 2018 and 2019. JSSU.

[33] Hou W, Chen S, Zhu C, Gu Y, Zhu L, Zhou Z (2023). Associations between smoke exposure and osteoporosis or osteopenia in a US NHANES population of elderly individuals. Front Endocrinol.

[34] Weng W, Li H, Zhu S (2022). An overlooked bone metabolic disorder: cigarette smoking-Induced osteoporosis. Genes.

[35] Wang J, Shu B, Tang DZ, Li CG, Xie XW, Jiang LJ, Jiang XB, Chen BL, Lin XC, Wei X, Leng XY, Liao ZY, Li BL, Zhang Y, Cui XJ, Zhang Q, Lu S, Shi Q, Wang YJ (2023). The prevalence of osteoporosis in China, a community based cohort study of osteoporosis. Front Public Health.

[36] Zamani M, Zamani V, Heidari B, Parsian H, Esmaeilnejad-Ganji SM (2018). Prevalence of osteoporosis with the World Health Organization diagnostic criteria in the Eastern Mediterranean Region: a systematic review and meta-analysis. Arch Osteoporos.

[37] Irani AD, Poorolajal J, Khalilian A, Esmailnasab N, Cheraghi Z (2013). Prevalence of osteoporosis in Iran: a meta-analysis. J Res Med Sci.

[38] Marques EA, Elbejjani M, Gudnason V, Sigurdsson G, Lang T, Sigurdsson S (2018). Cigarette smoking and hip volumetric bone mineral density and cortical volume loss in older adults: the AGES-Reykjavik study. Bone.

[39] Heydari Z, Shahesmaeili A, Khajeh-Bahrami MR, Rezazadeh-Mehrizi M, Gozashti MH, Moazed V (2017). An investigation of the risk factors of osteoporosis and the correlation between Opium Consumption and osteoporosis in adults. Addict Health.

[40] Cheraghi Z, Doosti-Irani A, Almasi-Hashiani A, Baigi V, Mansournia N, Etminan M, Mansournia MA (2019). The effect of alcohol on osteoporosis: a systematic review and meta-analysis. Drug Alcohol Depend.

[41] Ke Y, Hu H, Zhang J, Yuan L (2023). Alcohol consumption and risk of fractures: a systematic review and dose-response Meta-analysis of prospective cohort studies. Adv Nutr.

[42] Sommer I, Erkkila AT, Jarvinen R (2013). Alcohol consumption and bone mineral density in elderly women. Public Health Nutr.

[43] Cui Z, Meng X, Feng H, Zhuang S, Liu Z, Zhu T, Ye K, Xing Y, Sun C, Zhou F (2019). Estimation and projection about the standardized prevalence of osteoporosis in mainland China. Arch Osteoporos.

[44] Chen P, Li Z, Hu Y (2016). Prevalence of osteoporosis in China: a meta-analysis and systematic review. BMC Public Health.

[45] Ko CH, Yu SF, Su FM, Chen JF, Chen YC, Su YJ, Lai HM, Chiu WC, Hsu CY, Cheng TT (2018). High prevalence and correlates of osteoporosis in men aged 50 years and over: a nationwide osteoporosis survey in Taiwan. Int J Rheum Dis.

[46] Schmidt T, Schmidt C, Strahl A, Mussawy H, Rolvien T, Jandl NM (2020). A system to determine risk of osteoporosis in patients with Autoimmune Hepatitis. Clin Gastroenterol Hepatol.

[47] Kobza AO, Herman D, Papaioannou A, Lau AN, Adachi JD (2021). Understanding and managing corticosteroid-Induced osteoporosis. Open Access Rheumatol.

[48] Laurent MR, Goemaere S, Verroken C et al. Prevention and treatment of glucocorticoid-induced osteoporosis in adults: consensus recommendations from the Belgian bone club. Frontiers in endocrinology. 2022;13:908727. 10.3389/fendo.2022.908727 PMC9219603 35757436

[49] Tanaka Y (2021). Managing osteoporosis and joint damage in patients with rheumatoid arthritis: an overview. J Clin Med.

[50] Tong X, Chen X, Zhang S (2019). The Effect of Exercise on the Prevention of osteoporosis and bone angiogenesis. Biomed Res Int.

[51] Lee JH, Kim JH, Hong AR, Kim SW, Shin CS (2020). Optimal body mass index for minimizing the risk for osteoporosis and type 2 diabetes. Korean J Intern Med.

[52] Pinheiro MB, Oliveira J, Bauman A (2020). Evidence on physical activity and osteoporosis prevention for people aged 65 + years: a systematic review to inform the WHO guidelines on physical activity and sedentary behavior. Int J Behav Nutr Phys Act.

[53] Brooke-Wavell K, Skelton DA, Barker KL (2022). Strong, steady and straight: UK consensus statement on physical activity and exercise for osteoporosis. Br J Sports Med.

